# Validation of a Simple Score for Mortality Prediction in a Cohort of Unselected Emergency Patients

**DOI:** 10.1155/2022/7281693

**Published:** 2022-09-23

**Authors:** Jeannette-Marie Busch, Isabelle Arnold, John Kellett, Mikkel Brabrand, Roland Bingisser, Christian H. Nickel

**Affiliations:** ^1^Emergency Department, University Hospital Basel, University of Basel, Basel, Switzerland; ^2^Department of Emergency Medicine, Hospital of South West Jutland, Esbjerg, Denmark; ^3^Department of Emergency Medicine, University of Southern Denmark, Odense, Denmark

## Abstract

**Background:**

Prognostication is an important component of medical decision-making. A patients' general prognosis can be difficult to measure. The Simple Prognostic Score (SPS) was designed to include patients' age, mobility, aggregated vital signs, and the treating physician's decision to admit to aid prognostication. *Study Aim*. Our study aim is to validate the SPS, compare it with the Emergency Severity Index (ESI) regarding its prognostic performance, and test the interrater reliability of the subjective variable of the decision to admit.

**Methods:**

Over a period of 9 weeks all patients presenting to the ED were included, routinely interviewed, final disposition registered, and followed up for one year. The C-statistics of discrimination was used to compare SPS and ESI predictions of 7-day, 30-day, and 1-year mortality. Youden J Statistics and Odds ratio, using logistical regression, were calculated for the Simple Prognostic Score. In a subset, a chart review was performed by senior physicians for a secondary assessment of the decision to admit. Interrater reliability was calculated using percentages and Cohens Kappa.

**Results:**

Out of 5648 patients, 3272 (57.9%) had a low SPS (i.e., ≤ 1); none of these patients died within 7 days, 2 (0.1%) died within 30 days after presentation and 19 (0.6%) died within a year. The area under the curve for 1-year mortality of the Simple Prognostic Score was 0.848. Secondary analysis of the interrater agreement for the decision to admit was 92%.

**Conclusion:**

In a prospective study of unselected ED patients, the Simple Prognostic Score was validated as a reliable predictor of short- and long-term mortality.

## 1. Introduction

Patients wish to be informed about their prognosis and the likely course of their illness [1–3] to define personal healthcare goals [4] and evaluate the benefits and risks [5] of diagnostic investigations and treatment. Prognostication, therefore, is fundamental for informed shared decision-making [5–7] and an important component [8] of many areas of medical practice including emergency medicine.

There are several approaches to prognostication. Disease-specific scores [9, 10] have been developed to assess prognosis regarding specific disease states. Complex scores, such as the Acute Physiology and Chronic Health Evaluation (APACHE), which needs 34 parameters including laboratory results [11], would be difficult to implement and impractical to use in the ED. The surprise question, a subjective estimation commonly used in palliative oncological patients [12], has been found to be associated with patients' mortality in the emergency department [13]. Age [8], comorbidities [14], and frailty status in patients aged 65 and older [15] have been identified as patient characteristics with prognostic value. While these scores, subjective estimation, and patient characteristics can be used to evaluate prognosis in specific patients and settings, we could not identify a validated score only assessing prognosis in unselected patients in the emergency department.

Although triage scores [16] may contain prognostic properties, their primary purpose is to determine acuity and help prioritize patients. Therefore, patients with an excellent prognosis, such as those with severe renal colic, should be assigned high acuity levels [17]. Nevertheless, there will also always be a need to independently estimate the patient's likely prognosis to ensure wise and appropriate management decisions are made.

The simple prognostic score assesses 7-day, 30-day and 1-year mortality in unselected patients. It is based on age, vital signs, mobility, and the clinical decision to admit to hospital [7]. It was derived from a cohort of undifferentiated emergency patients presenting to two EDs in Denmark and Switzerland [18].

In this study, we aimed to validate the SPS in an independent cohort of all unselected patients presenting to the ED. We compared the predictive performance of the SPS with the Emergency Severity Index (ESI), a well-established triage tool and predictor of adverse outcomes. We also tested the interrater reliability for the subjective variable of the decision to admit, comparing the original disposition to an adjudicated admission decision.

## 2. Methods

### 2.1. Study Design and Setting

A secondary analysis of a prospective single-center all-comer observational study was conducted for quality control in the University Hospital of Basel Emergency Department, a tertiary care center with approximately 50,000 annual ED visits.

### 2.2. Data Collection

Every patient presenting to the Emergency Department (ED) of the University Hospital of Basel between March 18^th^, 2019 and May 20^th^, 2019 was included by a trained study team. All patients were triaged 24 hours a day, 7 days a week by a trained emergency nurse or physician using the ESI [16]. The need for written consent was waived due to the observational character of the study, and all patients that did not explicitly decline the study participation were included. Patients were then approached by study personnel for a short interview and routine vital sign measurements, such as heart rate, blood pressure, level of consciousness (as determined by the ACVPU scale: alert/new/confusion/verbal/pain/unresponsive), body temperature, peripheral oxygen saturation, and respiratory rate. Baseline characteristics such as age, sex, and diagnosis at ED discharge were obtained from the electronic health record (EHR) provided by PROTEC Data®, Boswil, Switzerland.

We conducted 2 separate structured health record reviews. Both were in accordance with 7 of the 8 points Gilbert et al. [19] listed as relevant for record review, as not all reviewers were blinded to the study outcomes. The first chart review was conducted for missing data by three reviewers; if the first two reviewers disagreed, a third independent reviewer adjudicated. Only presentations with missing data to calculate the National Early Warning Score (NEWS) and to assess Mobility On Presentation (MOP) were selected for review.

The second chart review was conducted for the secondary assessment of the decision to admit. Two senior physicians, with 25–30 years of clinical experience, reviewed 100 randomly selected patient presentations and assessed, based on information in the patient chart, if hospital admission was required or not. If there was disagreement between these two experts, the presentation was discussed by the full study team and an adjudicated decision was determined.

### 2.3. Follow-Up

Patients were followed up for mortality by query of the official registry of Switzerland, if they had a valid Swiss social security number. If this was not applicable, the patient was checked for repeated presentation after one year or contacted by phone. If not contactable by phone, proxies or primary care providers (PCP) were contacted either in person or in writing. If no information could be retrieved, a patient was classified as lost to follow-up.

### 2.4. Patient Selection

To validate the SPS, we only included records of patients who were alert according to the ACVPU Score and who had complete data on NEWS, MOP, and follow-up. As the protocol allowed repeated presentations, only the first ED presentation of a patient was included in the study to ensure a correct calculation of mortality rates.

### 2.5. Study Aims

The primary aim of this analysis is the validation of the SPS to predict mortality up to one year after presentation to ED [18].

The secondary aims of this study were comparison of the predictive performance of SPS with the ESI, and the interrater reliability of the decision to admit patients to the hospital.

### 2.6. Statistical Methods

The software used for the analysis was R Studios version 4.1.1 (https://www.Rproject.org/). Numeric variables were compared using the Student's *t*-test, and categorical variables were compared using Chi square analysis when applicable.

We calculated and compared the Receiver Operating Characteristics (ROC) and its Area Under the Curve (AUC) for the SPS and ESI for 1-year, 30 days, and 7 days mortality according to the method of Hanley and McNeil [20].

Sensitivity and specificity were calculated for the 1-year mortality of the SPS of ≥2 points, as well as the Youden statistic [21] and the odds ratio (OR) using multivariable logistic regression.

Interrater reliability of the decision to admit a patient to the hospital was determined by comparing an adjudicated admission decision with the actual decision made using percentages and Cohen's unweighted kappa. Cohen's unweighted kappa is a coefficient used to show the rate of agreement between raters ranging from 0.0 (no agreement) to 1.0 (complete agreement), taking into account the possibility of agreement due to chance [22].

### 2.7. Ethics

This study was approved by the local ethics committee (identifier 236/13, ww.eknz.ch, amendment for prolongation PB_2019_00008) and conducted according to the principles of the Declaration of Helsinki. The written informed consent was waived due to the observational nature of the study. Patients were excluded if the EHR contained a general rejection to participation in research or if they actively declined participation.

## 3. Results

**Figure 1:**
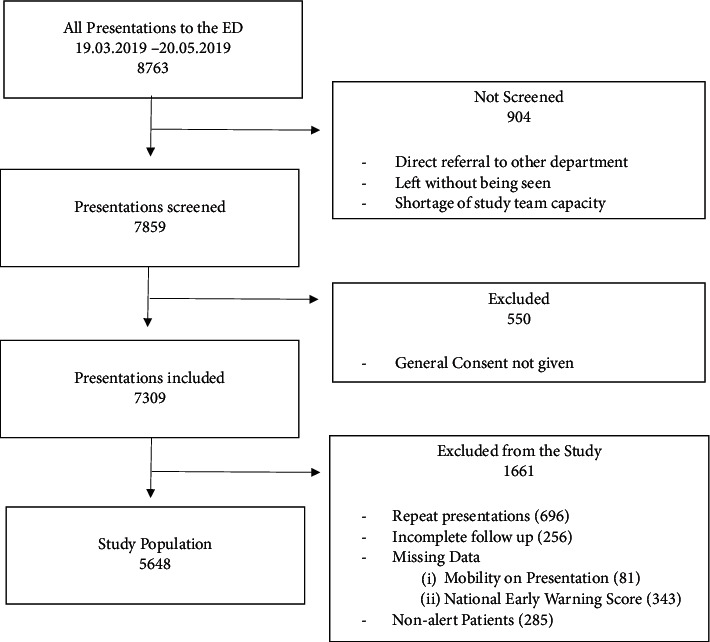
Recruitment and inclusion procedure for study population: This figure shows the recruitment and inclusion/exclusion procedure of the study population (ED = emergency department).

**Figure 2:**
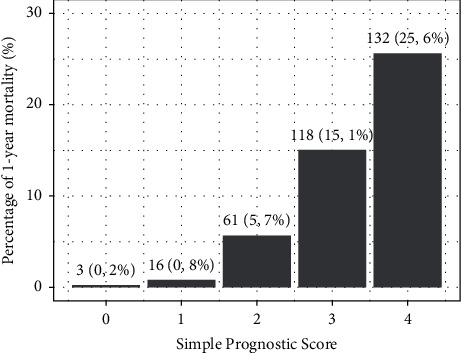
1-year mortality rate for each point of the Simple Prognostic Score (SPS). This figure shows the percentage of 1-year mortality for each point of the SPS.

Out of 7309 ED presentations, 1661 (22.7%) had to be excluded due to missing data (incomplete NEWS, IMOP, ACVPU, follow-up), being nonalert, or beinga repeat presentation (Figure 1). The excluded patients had the same age and sex as the included patients. However, they differed in level of acuity, mean NEWS, as well as MOP (Table 1). Of the final study population of 5648 patients, 3272 (57.9%) had none or only one SPS predictor (Figure 2); only 19 (0.6%) of these patients died within one year, 2 (0.1%) within 30 days, and none (0%) within 7 days. Of the 2376 (42.1%) of patients with a SPS of two or more, 311 (13.1%) died within a year. Of the 311 patients with a score of ≥2 who died within a year, 56 (18.0%) were discharged from the ED (Tables 2 and 3).

**Figure 3:**
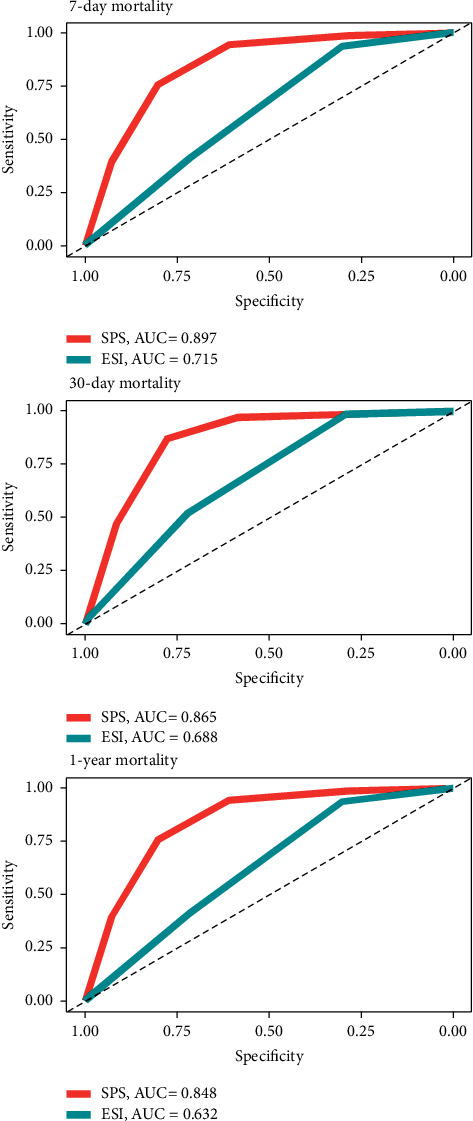
Receiver operating characteristic (ROC) curves. This figure shows ROC curves for emergency severity index (ESI) and the simple prognostic score (SPS) for 7-day, 30-day, and 1-year mortality. The ESI level was coded inversely for the ROC curve to lie above the diagonal. SPS, simple prognostic score,; ESI, emergency severity index; AUC, area under the curve.

The SPS had an AUROC of 0.848 for 1-year mortality, 0.865 for 30-day mortality, and 0.897 for 7-day mortality. In comparison, the AUROC of the ESI for mortality was significantly lower regarding all timespans (Figure 3, Table 4, Supplemental Figure 1).

An SPS ≥1 had the highest sensitivity and lowest specificity and positive and negative likelihood ratios, whereas 4 points had the lowest sensitivity and highest specificity and positive and negative likelihood ratios. However, there was almost no difference between the Youden J statistics for an SPS ≥2 and ≥ 3 points (i.e., 0.55 versus 0.56), unlike the sensitivity (i.e., 0.94 versus 0.76) (Table 5, Supplemental Table 1).

When corrected for age and sex, the odds ratio for death within a year for SPS ≥2 points is 7.3 (CI 4.5–12.4). For every point on the SPS added, the odds ratio for a patient to die within the next year would be higher by 2.0 (CI 1.8–2.2).

The agreement between the observed hospital admission decision and the adjudicated one was 92%, which had an interrater reliability kappa score of 0.60 (95% CI 0.42–0.77).

## 4. Discussion

This prospective validation study of the SPS in a large and unselected cohort of alert adult patients showed that the SPS predicts short- and long-term mortality more accurately than the ESI triage tool. Moreover, the decision for hospital admission, one subjective component of the score, has an acceptable level of interrater agreement based on a retrospective electronic health record review.

Prognostication is fundamental in the ED setting for several reasons. First, the prognosis is relevant for patient safety [23], identifying the necessity of urgent diagnostics or treatments of admission or discharge and thus potentially reducing risks of admission-delirium, falls or hospital acquired infections [24], or the low risk of unanticipated death after discharge [25–27]. Second, diagnostic work-up and treatment decisions, such as the need for observation, hospital admission, or discharge as well as the follow-up strategy should be determined with consideration to the overall prognosis and potential benefit and risk for the patient. Third, an evidence-based prognosis is the foundation for shared decision-making and individualized care [8, 28–30].

While life expectancy can be calculated for a certain population, it is more difficult to ascertain for an individual. The “surprise question,” developed for the assessment of the need for palliative care in oncological patients [12], was recently investigated for the association with 1-month mortality among undifferentiated older patients in the ED. That study found that while the odds of death were 2.4-fold higher if clinicians answered they would not be surprised if the patient died in the next month, the sensitivity is inadequate. This shows that a subjective assessment as a means of mortality prediction is imperfect [13]. However, our validation shows it is possible to estimate the risk of death for up to one year using the SPS, making an assessment of 1-year mortality more comprehensible concept and the SPS a possible tool in the assessment and communication of the potential risk [31].

The SPS reliably predicts both short- and long-term mortality. Patients with a high SPS are more likely to die within a year and should therefore be considered for admission, monitoring, or careful discharge planning with close follow up, depending on patient wishes and specific care needs. On the other hand, patients with a low SPS are at a low risk of death within a year and could be considered, after careful clinical evaluation, for early discharge and scheduled out-patient investigations if indicated.

Of the patients discharged with an SPS ≥2 who died within the follow-up period, a high proportion were frail older adults who were institutionalized and/or receiving palliative care. Therefore, prognosis alone does not necessarily warrant hospital admission. Nevertheless, an objective estimate of prognosis is needed to make wise and appropriate management decisions tailored to the patients' specific situation and needs. The SPS could be used to enhance communication in shared decision-making, helping to balance treatment risks, costs, and benefits against the patients' personal goals and values.

The interrater agreement for the decision of hospital admission is acceptable. While it has been shown, that the first visual assessment of a patient by a physician can already predict disposition fairly accurately (77%) [32] and is noninferior to structured triage [33], the final disposition decision is influenced by numerous factors [34]. These factors could explain different disposition decisions by different physicians for the same patients. Nonmedical factors influencing the treating physician can be physician-specific, patient-specific, or institutional. Physician-specific factors include patient load at presentation time [35]—increasing number of patients also increasing admission, risk preference [36, 37]—admission is more likely the more risk-adverse the physician is, and individual experience [32]. Patient-specific factors include living situation (e.g., children at home) [38, 39], substance abuse [40]—patients are less likely to agree to admission, and established and well managed out-patient care or simply the patients [41] or proxies' preference [42]. Institutional factors include hospital capacity and inadequate out-patient care [34], necessitating inpatient care.

### 4.1. Limitations

This study was performed in a single center, which had also participated in the derivation of the original score. Only 256 (3.5%) patients were lost to one-year follow-up.

As the decision to admit of the treating physician was not documented, the final disposition was used in lieu. This could increase the SPS for individual patients by one point, e.g., patients who left against medical advice. It could also limit the significance of the interrater reliability, as we compare the decision to admit of our reviewers with the final disposition of the treating physician, which is influenced by administrative classification and by patients' or proxies' preferences and does not represent the treating physicians' decision on its own. The IRR of MOP has, to the best of our knowledge, has not been tested and no exploration of confounding factors have been conducted.

## 5. Conclusion

In this prospective study of unselected ED patients, the Simple Prognostic Score was validated as a reliable predictor of short- and long-term mortality. It takes little extra effort and could be a useful tool to aid physicians making difficult decisions for alert and calm patients, such as decisions on disposition.

## Figures and Tables

**Table 1:** Demographics of included and excluded presentations Data are shown as mean and SD for continuous variables and as count and percentage for categorical variables.

	Excluded (*n* = 1661)	Included (*n* = 5648)	SMD	*P*
Age (mean (SD))	54.2 (22.3)	53.3 (21.6)	0.041	0.134
Sex (*n* (%) female)	734 (44.2)	2685 (47.5)	0.067	0.017
Emergency Severity Index (*n* (%))			0.350	<0.001
(i) 1	(i) 116 (7.0)	(i) 51 (0.9)		
(ii) 2	(ii) 483 (29.1)	(ii) 1540 (27.3)		
(iii) 3	(iii) 614 (37.0)	(iii) 2422 (42.9)		
(iv) 4	(iv) 395 (23.8)	(iv) 1544 (27.3)		
(v) 5	(v) 52 (3.1)	(v) 89 (1.6)		
(vi) NA	(vi) 1 (0.0)	(vi) 2 (0.0)		
Mortality (*n* (%))				
(i) In hospital	*†*	(i) 33 (0.6)	0.192	
(ii) 1 day		(ii) 7 (0.1)	0.163	
(iii) 7 day		(iii) 25 (0.4)	0.232	
(iv) 30 day		(iv) 69 (1.2)	0.261	
(v) 100 day		(v) 152 (2.7)	0.252	
(vi) 1 year		(vi) 330 (5.8)	0.258	
Hospital admission (*n* (%))	686 (41.3)	1917 (33.9)	0.152	<0.001
NEWS (mean (SD))	2.67 (3.06)	1.27 (1.71)	0.561	<0.001
IMOP (%)	636 (38.3)	1444 (25.6)	0.323	<0.001

IMOP, impaired mobility on presentation; NEWS, National early warning score; SMD, standardized mean difference; SD, standard deviation. *†*as the excluded group included repeat presentations, mortality could not be calculated.

**Table 2:** Demographics of patients stratified by the SPS of ≥2. Data are shown as mean and SD for continuous variables and as count and percentage for categorical variables.

	<2 (*n* = 3272)	≥2 (*n* = 2376)	SMD	*P*
Age (mean (SD))	42.3 (16.0)	68.3 (18.9)	1.480	<0.001
Sex (*n* (%) female)	1517 (46.4)	1168 (49.2)	0.056	0.040
Emergency Severity Index (*n* (%))			0.999	<0.001
(i) 1	(i) 1 (0.0)	(i) 50 (2.1)		
(ii) 2	(ii) 532 (16.3)	(ii) 1008 (42.4)		
(iii) 3	(iii) 1290 (39.4)	(iii) 1132 (47.6)		
(iv) 4	(iv) 1364 (41.7)	(iv) 180 (7.6)		
(v) 5	(v) 83 (2.6)	(v) 6 (0.3)		
(vi) NA	(vi) 2 (0.0)	(vi) 0		
Mortality (*n* (%))				
(i) In hospital	(i) 0 (0)	(i) 33 (1.4)	0.168	<0.001
(ii) 1 day	(ii) 0 (0)	(ii) 7 (0.3)	0.077	0.006
(iii) 7 day	(iii) 0 (0)	(iii) 25 (1.1)	0.146	<0.001
(iv) 30 day	(iv) 2 (0.1)	(iv) 67 (2.8)	0.233	<0.001
(v) 100 day	(v) 7 (0.2)	(v) 145 (6.1)	0.342	<0.001
(vi) 1 year	(vi) 19 (0.6)	(vi) 311 (13.1)	0.512	<0.001
Hospital admission (*n* (%))	177 (5.4)	1740 (73.2)	1.929	<0.001
NEWS (mean (SD))	0.70 (1.08)	2.06 (2.08)	0.823	<0.001
IMOP (*n* (%))	115 (3.5)	1329 (55.9)	1.397	<0.001

IMOP, impaired mobility on presentation; NEWS, National early warning score; SMD, standardized mean difference; SD, standard deviation.

**Table 3:** 1-year mortality according to the SPS and admission. 1-year mortality according to the SPS and hospital admission, also showing the total number of mortality per score group.

			1-year mortality							
Score	Total		Admitted (%)		Admitted (%)		Discharged (%)		All (%)	
0	1326	(23.5)	0	(0)	—	—	3	(0.2)	3	(0.2)
1	1946	(34.5)	177	(9.1)	2	(1.1)	14	(0.8)	16	(1.3)
2	1077	(19.1)	554	(51.4)	31	(5.6)	30	(5.7)	61	(5.7)
3	784	(13.9)	671	(85.6)	92	(13.7)	26	(23.0)	118	(15.0)
4	515	(9.1)	515	(100)	132	(25.6)	—	—	132	(25.6)
Total	5648	(100.0)	1917	(33.9)	257	(13.4)	73	(2.0)	330	(5.8)

SPS simple prognostic score.

**Table 4:** Comparison of the AUROC of SPS and ESI. Comparison of the area under the curve of the receiving operating characteristics of the Simple Prognostic Score and Emergency Severity Index.

	AUC SPS (CI 95%)	AUC ESI (CI 95%)	*P*-value
7-day mortality	0.897 (0.863–0.931)	0.715 (0.646–0.784)	0.011
30-day mortality	0.865 (0.833–0.898)	0.688 (0.642–0.734)	<0.001
1-year mortality	0.848 (0.831–0.865)	0.632 (0.608–0.656)	<0.001

SPS, simple prognostic score; ESI, emergency severity index; AUROC, area under the curve of the receiving operating characteristics; AUC, area under the curve.

**Table 5:** Predictive characteristics of the SPS. Predictive characteristics for each possible point threshold of the simple prognostic score, with the chosen threshold of ≥2. Corresponding contingency tables can be found in the online supplement.

	≥1	CI 95%	≥2	CI 95%	≥3	CI 95%	≥4	CI 95%
True prevalence	0.06	0.05, 0.06	**0.06**	**0.05, 0.06**	0.06	0.05, 0.06	0.06	0.05, 0.06
Sensitivity	0.99	0.97, 1	**0.94**	**0.91, 0.96**	0.76	0.71, 0.80	0.4	0.35, 0.46
Specificity	0.25	0.25 0.26	**0.61**	**0.6, 0.62**	0.80	0.79, 0.81	0.93	0.92, 0.93
PPV	0.08	0.07 0.08	**0.13**	**0.12, 0.15**	0.19	0.17, 0.21	0.26	0.22, 0.30
NPV	1	0.99, 1	**0.99**	**0.99, 1**	0.98	0.98, 0.99	0.96	0.96, 0.97
PLR	1.32	1.29, 1.34	**2.43**	**2.32, 2.53**	3.84	3.54, 4.17	5.55	4.72, 6.54
NLR	0.04	0.01, 0.11	**0.09**	**0.06, 0.15**	0.30	0.25, 0.37	0.65	0.59, 0.71
Youden J	0.24	0.22, 0.26	**0.55**	**0.51, 0.58**	0.56	0.50, 0.61	0.33	0.27, 0.39

PPV, positive predictive value; NPV, negative predictive value; PLR, positive likelihood ratio; NLR, negative likelihood ratio; J Youden Index.

## Data Availability

Due to patient privacy, data cannot be made available.

## Supplementary Materials

Supplementary Materials Supplemental Figure 1: summary of Simple Prognostic Score and Emergency Severity Index. This figure shows the values of the SPS broken down by ESI. Supplemental Table 1: contingency tables for the predictive characteristic of each possible threshold of the SPS.
