# Supplementary material for: Validation of a Simple Score for Mortality Prediction in a Cohort of Unselected Emergency Patients
Source: Int J Clin Pract. 2022 Sep 23;2022:7281693. doi: 10.1155/2022/7281693 (PMC9525775; doi:10.1155/2022/7281693)
Supplement: Supplementary Materials — Supplemental Figure 1: summary of Simple Prognostic Score and Emergency Severity Index. This figure shows the values of the SPS broken down by ESI. Supplemental Table 1: contingency tables for the predictive characteristic of each possible threshold of the SPS. [file 7281693.f1.docx]

Supplement

| Supplemental Table 1: Contingency Tables  Contingency Tables for the predictive characteristic of each possible threshold of the SPS | | |
| --- | --- | --- |
| SPS | Dead | Alive |
| 0 | 3 | 1323 |
| ≥ 1 | 327 | 3995 |
|  |  |  |
| ≤ 1 | 19 | 3253 |
| ≥ 2 | 311 | 2065 |
|  |  |  |
| ≤ 2 | 80 | 4269 |
| ≥ 3 | 250 | 1049 |
|  |  |  |
| ≤ 3 | 198 | 4935 |
| 4 | 132 | 383 |
| *SPS Simple Prognostic Score | | |

**Supplemental Figure 1: Summary of Simple Prognostic Score and Emergency Severity Index**


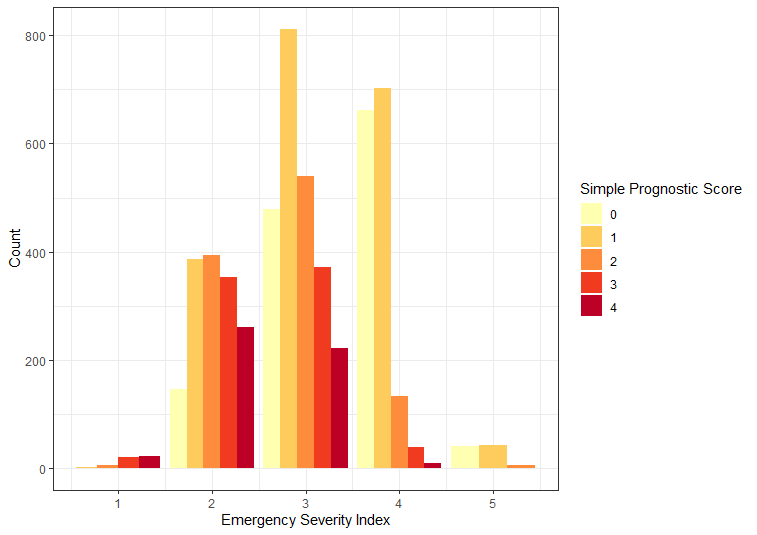
This figure shows the values of the SPS broken down by ESI
